# An Ethical Review of Euthanasia and Physician-assisted Suicide

**Published:** 2017-02

**Authors:** Božidar BANOVIĆ, Veljko TURANJANIN, Anđela MILORADOVIĆ

**Affiliations:** 1. Faculty of Security Studies, University at Belgrade, Belgrade, Serbia; 2. Faculty of Law, University at Kragujevac, Kragujevac, Serbia; 3. Seniorenzentrum Röweland, Hamburg, Germany

**Keywords:** Active euthanasia, Passive euthanasia, Physician-assisted suicide, Ethics, Morality

## Abstract

**Background:**

In the majority of countries, active direct euthanasia is a forbidden way of the deprivation of the patients’ life, while its passive form is commonly accepted. This distinction between active and passive euthanasia has no justification, viewed through the prism of morality and ethics. Therefore, we focused on attention on the moral and ethical implications of the aforementioned medical procedures.

**Methods:**

Data were obtained from the Clinical Hospital Center in Kragujevac, collected during the first half of the 2015. The research included 88 physicians: 57 male physicians (representing 77% of the sample) and 31 female physicians (23% of the sample). Due to the nature, subject and hypothesis of the research, the authors used descriptive method and the method of the theoretical content analysis.

**Results:**

A slight majority of the physicians (56, 8%) believe that active euthanasia is ethically unacceptable, while 43, 2% is for another solution (35, 2% took a viewpoint that it is completely ethically acceptable, while the remaining 8% considered it ethically acceptable in certain cases). From the other side, 56, 8% of respondents answered negatively on the ethical acceptability of the physician-assisted suicide, while 33% of them opted for a completely ethic viewpoint of this procedure. Out of the remaining 10, 2% opted for the ethical acceptability in certain cases.

**Conclusion:**

Physicians in Serbia are divided on this issue, but a group that considers active euthanasia and physician-assisted suicide as ethically unacceptable is a bit more numerous.

## Introduction

Does a man, in addition to the right to live, have a right to die? More specifically, does he have a right to a dignified death? Is a deprivation of life from the mercy reasons a crime or unpunishable act? The answer to these questions varies from country to country. On the one hand, if a state decides to legalize this form of the deprivation of life, the key question is what are the reasons for it? On the other hand, in a case when legislator takes an opposite view, we have the same question (1). Therefore, euthanasia, and in recent time physician-assisted suicide, are inexhaustible topics for reflection and observation of the different aspects of medicine, law, sociology, philosophy, religion and morality (according to some authors, this debate is one of the ten hotly moral issues (2), but also one of the major problems in the national and international health limits. By bypassing defining these two very famous terms at this point in time, we will just point out that the direct active euthanasia is a medical act directed to the deprivation of life (hereinafter: ADE), while a physician-assisted suicide is an act of the physician where he provides to the patient a medicament for taking life (hereinafter: PAS).

It is not clear when the man for the first time came to the idea of euthanasia (3). There have been many discussions in the United States and United Kingdom, culminated in 1906, when Ohio attempted to pass a law to legalize euthanasia (4). Movements to the legalization of the ADE and PAS have marked the last few decades, but we can notice that legislators across the world more easily decriminalize PAS, as a milder form of the deprivation of life. This is primarily evident on the American continent, where a few states decriminalized PAS, although the Supreme Court held that there is no constitutional right to ADE and PAS, nor the ban on the mentioned acts. Parallel to this process, there are strict criticisms of such actions, which have the ultimate aim of eliminating criminal penalties for persons who assist in the deprivation of the life of the patient, who is terminally ill at his request (5). If we take the example of England, the constant change of the attitudes of the British Medical Association and Royal College of Physicians, that varies from the strict opposition to the neutral position and vice versa, shows that is hard for them to accept any attitude regarding taking one’s life and to take any constant attitude whatsoever (6).

Different viewpoints in some countries caused a different approach to the legislative treatment of these two issues (7, 8), but their solutions are, due to the many activities in this area, constantly reviewed (9). For example, Belgium in 2014 went far ahead when approved ADE for children, considering them as competent for such decision (10, 11). Both procedures are in the majority of countries in the world illegal, but they exist everywhere (12).

However, in this paper, we will mainly deal with the observation of the ADE and PAS from ethical point of view, where we devote due attention to the criticism of a different regulation of ADE and passive euthanasia (hereinafter: PE), which is inexhaustible field for everyone who seriously takes this matter. In order to contribute to existed theoretical and practical considerations, we conducted a survey among physicians in Serbia on this topic.

## Materials and Methods

The data for the current analysis have been derived from the broader research project whose aim was to identify occurrence, distribution, and opinions of the physicians about euthanasia and physician-assisted suicide. In this paper, we analyzed part of the obtained data. Research is primarily based on quantitative research approach, and data were collected using a short survey, created specifically for the purpose of this study. In the civilized countries, physicians are increasingly faced with demands to assist patients in committing suicide or to apply euthanasia (13, 14). Therefore, we conducted a survey among the physicians from Clinical Hospital Center in Kragujevac (Serbia) in 2015. Data were collected during the first half of the mentioned year. We analyzed the segments of dataset which concern to two questions: Which of them is acceptable: ADE ethically or PAS ethically? To both questions, we offered three answers: *yes*, *no*, and *yes, in some cases*.

The scope of the tested population, gender structure of the respondents, as well as the diversity of the health departments in participants employed, gives us possibility of a wider generalization of the findings to the physicians’ populations across the whole country. The initial sample plan was to try to conduct a survey of all employees in this medical institution. Of 100 physicians, 88 expressed their willingness to be participants. The final sample included 88 physicians: 57 male physicians (representing 64, 77% of the sample) and 31 female physicians (35, 23% of the sample). The study was divided into three parts: in the Ambulance, in the Emergency Room, while the third, the most numerous sample, and included physicians from the departments of Surgery, Transfusion, and Cardiology. The initial hypothesis was that the physicians who work in the Emergency Room are prone to saving lives, and will be exclusively against ADE and PAS. The same situation is expected in the Ambulance, while in the remaining sample, physicians will be divided by their opinions. We analyzed total data as well as data by departments.

## Results

The sample included 88 physicians, who declared on ethical acceptability of ADE and PAS. In Table 1 are total data for the ADE, while in the Table 2 are results located by departments; in the Table 3 are total data for the ethical acceptability for the PAS, and in the Table 4 are located data by departments for this question.

**Table 1:** Is ADE ethically acceptable?

		**Frequency**	**Percent**	**Valid Percent**	**Cumulative Percent**
Valid	Yes	31	35.2	35.2	35.2
	No	50	56.8	56.8	92.0
	Yes, in some cases	7	8.0	8.0	100.0
	Total	88	100.0	100.0	

**Table 2:** Is ADE ethically acceptable?

		**Institution**			**Total**

		**Ambulance**	**Other Departments**	**Emergency Room**	
	Yes	6 (31.5%)	25 (50%)	0	31
	No	12 (63.15%)	20 (40%)	18 (94.73%)	50
	Yes, in some cases	1 (5.35%)	5 (10%)	1 (5.27%)	7
Total		19	50	19	88

**Table 3:** Is PAS ethically acceptable?

		**Frequency**	**Percent**	**Valid Percent**	**Cumulative Percent**
Valid	Yes	29	33.0	33.0	33.0
	No	50	56.8	56.8	89.8
	Yes, in some cases	9	10.2	10.2	100.0
	Total	88	100.0	100.0	

**Table 4:** Is PAS ethically acceptable?

		**Institution**			**Total**

		**Ambulance**	**Other Departments**	**Emergency Room**	
	Yes	6 (31.5%)	23 (46%)	0	29
	No	12 (63.15%)	20 (40%)	18 (94.73%)	50
	Yes, in some cases	1 (5.35%)	7 (14%)	1 (5.27%)	9
	Total	19	50	19	88

## Discussion

The issue of the right to death with dignity is inevitably linked with ethics and morals. The law and moral in some cases does not stand in the necessary pervasive connection, due to the brutal features of some legal systems, although such phenomena should be as rare as possible (15).

In the literature, we can find another significant question: could the moral and ethical conduct be illegal, but the act to be in accordance with the law to be immoral (16)? If we start from the basic rule that the law is only a minimum of morality, thus the moral rules are at the higher level then legal. Based on this, when a legislator regulates ADE and PE, he should not make distinction between them, because they are equal in weight. In the numerous issues raised in the area of euthanasia, the existence or non-existence of moral differences between killing and letting to die a patient from the effects of the disease, and between ADE and PAS stands out (17).

The supporters of this attitude (most commonly in the sphere of philosophy and religion (18)) found one of the main reasons for the immorality of ADE in the assertion that human life is sacred (although this term usually belongs to the religion -“traditional ethical principle” (19, 20) that one cannot and must not take. They bypass a debate about the reasons that led to killing by using ADE. They do not put an accent on the quality and content of life. At the same time, these authors are divided into two fractions: radical and moderate. The supporters of moderate direction reject possibility of the moral justification and legal regulation of ADE, while the authors who hold the radical position are absolutely against any form of euthanasia and taking of human life. Human life is sacred and it is the work of the God (21–23). However, human life has a meaning only in a case when the brain is operational (24), and from the times of Confucius, we have had philosophical thought that biological life has not more value of the man (25). The supporters of ADE and PAS do not believe that these actions are immoral *per se*, especially in the situations where the patient is suffering from great pain (26). In the medical practice, we have such conditions of the patient in which their pain cannot be controlled or reduced. According to the specific research, in the best scenario, 97% of all pain can be brought under control, but 3% of pains remain and that can be unbearable and cannot be controlled (27). We have noted just one of the many examples of unreasonableness of the observation of euthanasia as an immoral procedure (28). Simply, the interests and the will of the patient should be above the wishes of doctors, and even legislators. According to some authors, respect for human being involves four dimensions: concern for his well-being, respect for his wishes, respect of the core values of his life and respect of his interests (29).

When a physician determines that the patient suffers from an incurable disease, death becomes the inevitable outcome, and therefore, we cannot seek the cause of death in the medical treatment, but in the natural reasons. Regardless of the fact that determines the patient’s life by his active engagement, his act cannot be considered as a direct cause of the patient’s death. This removes the doctor’s responsibility, legal and moral (30). The fact that lies in the basic of the ADE and PE is the intention to terminate a patient’s life, elaborated in the acting/omission doctrine. Some authors assert this view, stating that a big difference exists between deprivations of life and letting someone to die, citing the example of the hunger in the poor regions of the world. If we accept a view that there are no differences between ADE and PE, and arguing that persons who die from the hunger did not die from poverty and shortages of the food, and in that case, we are all killers (31). This attitude is unacceptable for the obvious reasons. It is very important difference between occurrence of a death as an effect of the direct physicians’ act and its occurrence as a side effect of the drug given with the aim of relieving pain (32). Obviously, it gives moral justifications primarily to the active indirect euthanasia, while ADE remains in every sense morally prohibited, which is the unsustainable structure. Here, it is also unacceptable to set up possibility of comparing these two modes of death, especially from the patient’s point of view. After the occurrence of death, the patient is indifferent about this issue (33).

The authors who argue about admissibility of legalization ADE and on its complete immorality and deny the possibility of the deletion of legal and moral dividing line, when presenting counter-arguments do not take into account, or they mention it only cursory, without deeper analysis, a crucial fact without no treatment applied – the will of the patient (31). Here, we bypass theories about autonomy of the will from the John Stuart Mill and Immanuel Kant, although some authors believe that the right to self-determination derives only from the teaching of Kant (2). Autonomy of the will is the basis of dignity of human nature and the every mind nature (34). However, the authors who are deeply involved in the studying of the individual autonomy of the will bring into the question the ability of the patient to express his will to ADE, because they are under the pressure to agree with such act (35). As a counter-argument, there is a possibility of abuse, but this is a case with every procedure in the world.

The main argument for the removal of the status of immorality from the ADE lies in the patient’s autonomy of the will, and not bypassed. Therefore, we ought once again to remind Dworkin’s thesis that the grossest form of the tyranny is allowing the death of the person in a manner that another people justify (27). At the same time, we have to bear in mind the best interest of the patient, not limited only to the perception of his physician (36). Based on that, we can assess the patient’s best interest, because, there are situations in life in which a patient wants shortening of his life despite the real possibilities of his healing, where one should restrict his autonomy of the will. In addition, a patient wants to continue his medical treatment, even though the doctor diagnosed that death is inevitable. In such cases, the autonomy of patient’s will have a dominant character. The application of the euthanasia on that patient would mean a violation of the all ethical principles that exist. The men who simply do not want to accelerate his death despite serious medical condition, either for religious or from nonreligious reasons, thus expressing their will that must be respected and their life must not be shortened by applying ADE. Nobody has a moral right to decide for another person whether his life is worth living or not, because, for one person his pain can be unbearable to the point that his life is of no value, while for others pain cannot be compared with the values of life. Any decision that patiently brings is morally acceptable for him. It must be the same for everyone else. Overall, from the Table 1, we can see that physicians were divided regarding the issue of the ethical acceptability of ADE. A slight majority, 56.8% believe that this method is ethically unacceptable, while 43.2% opted for another solution. In addition, 35.2% took a viewpoint that it is completely ethically acceptable, while the remaining 8% considered it ethically acceptable in certain cases. Respondents who viewed ADE ethically acceptable in certain situations could not deny its acceptability because they are aware of the fact that patient’s condition could be extremely difficult. This confirms initial hypothesis and we got expected results, not only here but also almost through the remaining results. Namely, in the region of Kragujevac, and also in the most part of Serbia, physicians did not yet meet with the ADE in practice, and therefore, their basic view on it and its ethical acceptability is mostly negative. Orthodox Church in the region contributes to such a view because it regards ADE and PAS as murders.

Our starting hypothesis is proved through the next, Table 2. The highest percentage of the respondents who declared themselves in favor of ethics ADE is among respondents in the third, the largest part of the sample. Of the 19 physicians in the Ambulance, six consider that this practice is ethically acceptable, while just one considered that it is ethically acceptable in some cases. Twelve physicians opted for the opposite response. In the Emergency Room, on the other hand, almost no one of the respondents did vote in favor of ethics, except one, who sees ADE as ethically acceptable in some cases. The results show the correctness of the assumption that the physicians who are in the first place turned to saving lives will be against ADE, and that physicians in the Ambulance with majority will be against ADE. Another part of the sample with the mild majority voted in favor of ADE - we expected that because this part of the sample is not on the front line of the struggle for the life of the patients. Precisely because of this group of respondents, the percentage of the physicians who are against the ethical acceptability of ADE does not deviate much from the supporters.

The following question tried to establish ethical acceptability of the PAS and results are shown in Table 3. What surprised us a bit is a greater support, even in a minuscule percentage, to the ADE in the relation to the PAS. As we can see, 56.8% of respondents answered negatively on the ethical acceptability of the PAS, while 33% of them opted for a completely ethic viewpoint of this procedure. Out of the remaining 10.2% opted for the ethical acceptability in certain cases. Although there are no excessive variations in relation to ADE, we assumed that a greater percentage of respondents would consider PAS more acceptable than ADE, since in this procedure physicians do not represent the main cause of the patient’s death. In PAS, they represent just accomplices, who will provide a necessary aid to the patients. In addition, physicians in some cases consider that they should not abandon their patients and that they should take responsibility for their death.

Results almost identically to the ADE are with PAS when we have a look at the distribution by departments in Table 4. The only difference is reflected in the fact that the number of respondents who believe that these procedures are ethical to have fallen from 25 to 23, while two respondents increased the number of those who believe that PAS is ethically acceptable in some cases. Moreover, we can assume that these are two same respondents. Therefore, if we compare the percentage of the subjects in the ADE and PAS tables in the relation to the Emergency Room, we have identical data. Simply, respondents are absolutely against these procedures, except for the one, who believes that in some cases PAS would be ethically acceptable. Absolute orientation to saving lives contributes their denial of the justification of any form of the deprivation of life.

## Conclusion

Euthanasia, regarded as deprivation of life with compassion, as well as PAS, is complex issues that cause and raise numerous questions. A particular problem is breaking euthanasia on ADE and PE, and then their different regulation. Morally and ethically, they are equal. In any case, autonomy of the will of the patient should be an essential moment. Physicians in Serbia are divided on this issue, but a group that considers ADE and PAS as ethically unacceptable is a bit more numerous. However, for the better view of their attitudes we should research on a much wider area.

## Ethical considerations

Ethical issues (Including plagiarism, Informed Consent, misconduct, data fabrication and/or falsification, double publication and/or submission, redundancy, etc) have been completely observed by the authors.
